# Antiviral Mechanism and Biochemical Basis of the Human APOBEC3 Family

**DOI:** 10.3389/fmicb.2012.00250

**Published:** 2012-07-09

**Authors:** Mayumi Imahashi, Masaaki Nakashima, Yasumasa Iwatani

**Affiliations:** ^1^Clinical Research Center, National Hospital Organization Nagoya Medical CenterNagoya, Japan; ^2^Graduate School of Medicine, Nagoya UniversityNagoya, Japan; ^3^Graduate School of Engineering, Nagoya UniversityNagoya, Japan

**Keywords:** antiviral, APOBEC3, APOBEC3G, cytidine deaminase, HIV, retrovirus, reverse transcription, Vif

## Abstract

The human APOBEC3 (A3) family (A, B, C, DE, F, G, and H) comprises host defense factors that potently inhibit the replication of diverse retroviruses, retrotransposons, and the other viral pathogens. HIV-1 has a counterstrategy that includes expressing the Vif protein to abrogate A3 antiviral function. Without Vif, A3 proteins, particularly APOBEC3G (A3G) and APOBEC3F (A3F), inhibit HIV-1 replication by blocking reverse transcription and/or integration and hypermutating nascent viral cDNA. The molecular mechanisms of this antiviral activity have been primarily attributed to two biochemical characteristics common to A3 proteins: catalyzing cytidine deamination in single-stranded DNA (ssDNA) and a nucleic acid-binding capability that is specific to ssDNA or ssRNA. Recent advances suggest that unique property of A3G dimer/oligomer formations, is also important for the modification of antiviral activity. In this review article we summarize how A3 proteins, particularly A3G, inhibit viral replication based on the biochemical and structural characteristics of the A3G protein.

## Introduction

Productive infections of primary human lymphocytes, monocytes, and certain T-cell lines by HIV-1 require a virally encoded gene product, Vif (originally named “Sor” or “A”; Fisher et al., 1987; Strebel et al., 1987). In early work on Vif, *vif*-deficient virions produced in non-permissive cells were found to be significantly impaired in their ability to complete reverse transcription (Sova and Volsky, 1993; von Schwedler et al., 1993), and they were 100- to 1000-fold less infectious than wild type (WT) virions (Fisher et al., 1987; Strebel et al., 1987; Fouchier et al., 1996). Sheehy et al. (2002) identified A3G as the cellular enzyme that restricts the replication of *vif*-deficient HIV-1.

The human A3G protein is a cellular cytidine deaminase that belongs to the APOBEC3 family, which comprises seven members (A3A, B, C, DE, F, G, and H; LaRue et al., 2009). These proteins contain one (A3A, A3C, and A3H) or two (A3B, A3DE, A3F, and A3G) zinc-cluster domains with the consensus sequence (H/C)XE(X)_23–28_CXXC (Wedekind et al., 2003). Among the APOBEC3 family members, A3G is the most potent inhibitor of HIV-1 but only in the absence of Vif. HIV-1 Vif counteracts A3G by promoting its polyubiquitination through the recruitment of a Cullin5-based E3 ubiquitin ligase complex (Yu et al., 2003), which targets A3G proteins for rapid proteasomal degradation in infected cells.

The specific A3G degradation is determined by the capability of Vif to bind with A3G in the E3 ubiquitin ligase complex (reviewed in Kitamura et al., 2011). The region in A3G responsible for HIV-1 Vif interaction was identified by the studies on the species specificity of Vif-mediated A3G degradation, which is determined by a single amino acid difference in human A3G, D128 versus K128 in the A3G of African green monkeys (Bogerd et al., 2004; Mangeat et al., 2004; Schrofelbauer et al., 2004; Xu et al., 2004). Subsequent mutational analyses have confirmed that the 128DPD130 motif of A3G, located near the zinc-coordinating residues of NTD, is crucial for direct binding to HIV-1 Vif (Huthoff and Malim, 2007; Russell et al., 2009; Lavens et al., 2010). This motif is just downstream of residues 124YYFW127, which are involved in A3G’s ability to bind nucleic acids (Huthoff and Malim, 2007).

The primary mechanism by which A3G inhibits *vif*-deficient HIV-1 replication requires its expression in virus producer cells and its incorporation into virions (Mariani et al., 2003; Marin et al., 2003; Sheehy et al., 2003; Stopak et al., 2003; Svarovskaia et al., 2004). During reverse transcription in the target cells, the virion-packaged A3G deaminates cytidine to uridine in the viral minus-strand DNA (Harris et al., 2003a; Lecossier et al., 2003; Mangeat et al., 2003; Zhang et al., 2003; Suspène et al., 2004; Yu et al., 2004). Subsequent incorporation of adenines instead of guanines in the plus-strand results in extensive G-to-A hypermutation and inactivation of the viral genome. Shortly after A3G was suggested as the key restriction factor against *vif*-deficient HIV-1, it was proposed that A3G-mediated deamination might be a lethal trigger, eventually leading to the degradation of reverse-transcribed viral DNA through a base-excision pathway and/or the reduced replication of progeny viruses by introducing premature stop codons and/or amino acid changes (Cullen, 2003; Goff, 2003; Harris et al., 2003a,b; KewalRamani and Coffin, 2003). Indeed, the catalytic center of the A3G protein is clearly essential for its antiviral functions (Mangeat et al., 2003; Navarro et al., 2005; Iwatani et al., 2006; Browne et al., 2009). However, several lines of recent evidence have indicated that the catalytic activity of A3G is not sufficient to explain its full antiviral activity. What is the fundamental mechanism(s) of A3G antiviral activity that explains the observation by von Schwedler et al. (1993), that the reverse transcription of *vif*-deficient HIV-1 is impaired when produced from A3G-expressing “non-permissive” cells?

## Biochemical Properties of A3G

**Figure 1:**
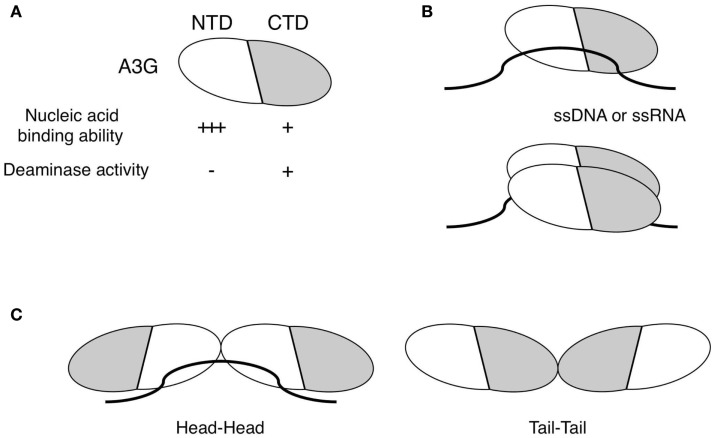
**Biochemical characteristics of A3G**. **(A)** A3G consists of an NTD and CTD. The NTD is responsible for the nucleic acid-binding affinity of A3G and has no detectable deaminase activity. In contrast, the CTD is solely involved in deaminase activity and has less affinity for nucleic acids than the NTD. **(B)** A3G specifically binds to single-stranded DNA (ssDNA) or RNA (ssRNA) but not to double-stranded nucleotides. A monomeric and/or dimeric A3G bind to ssDNA or ssRNA as the minimum unit. **(C)** The A3G protein forms homodimers or higher-order homo-oligomers through interactions between its NTDs (head–head) or CTDs (tail–tail).

The zinc coordination of A3 family proteins is mediated by a histidine and two cysteines, which form a catalytic center for cytidine deaminase activity. In A3G, the zinc-binding motif at the C-terminal domain (CTD) is primarily associated with cytidine deaminase catalysis whereas the N-terminal domain (NTD) does not catalyze deamination (Figure 1A; Haché et al., 2005; Navarro et al., 2005; Iwatani et al., 2006; Friew et al., 2009). The A3G enzyme converts deoxycytidine (dC) residues to deoxyuridine (dU), and acts preferentially on residues that are preceded by another dC, with a much higher preference for the 5′-CCCA-3′ sequence in single-stranded DNA (ssDNA; Beale et al., 2004; Suspène et al., 2004; Yu et al., 2004). During retroviral reverse transcription, A3G deaminates dC to dU in the viral minus-stranded DNA, and the subsequent incorporation of deoxyadenines (dA) instead of deoxyguanines (dG) in the plus-strand results in G-to-A hypermutation of the nascent viral DNA (Harris et al., 2003a; Lecossier et al., 2003; Mangeat et al., 2003; Zhang et al., 2003; Suspène et al., 2004; Yu et al., 2004). The ssDNA-specific deamination by A3G appears to be determined by a structural groove, presumably accommodating ssDNA, that positions the cytosine for deamination (Chen et al., 2008; Holden et al., 2008).

The nucleic acid-binding property of A3G is also a major biochemical feature. The minimum unit of A3G for binding to ssDNA is a monomer (Chelico et al., 2010) and/or a dimer (Bennett et al., 2008), as illustrated in Figure 1B. The apparent equilibrium dissociation constant (Kd) for A3G (to ssDNA) is between 52 and 238 nM (Chelico et al., 2006; Iwatani et al., 2006, 2007), whereas the Kd for HIV-1 nucleocapsid protein (NC) binding to RNA is approximately 23–320 nM (Shubsda et al., 2002; Levin et al., 2005), suggesting that the nucleic acid-binding affinity of A3G is as high as that of NC. A3G binds preferentially to ssDNA or ssRNA (Figure 1B; Yu et al., 2004; Iwatani et al., 2006; Shlyakhtenko et al., 2011), especially dT or dU residues of ssDNA and AU-rich regions in ssRNA, respectively (Jarmuz et al., 2002; Iwatani et al., 2006). Interestingly, the substrate specificities and nucleotide preferences of the A3G protein differ for its deaminase and nucleic acid-binding activities, as is the case for APO1 (Anant et al., 1995; Navaratnam et al., 1995; Anant and Davidson, 2000). Because mutations that disrupt zinc coordination at the NTD, such as the substitution of the C100 residue with a serine, abrogate the nucleic acid-binding affinity of A3G (Navarro et al., 2005; Iwatani et al., 2006), some local conformation near the zinc coordination of NTD might be responsible for its recognition of single-stranded nucleotides.

The formation of an A3G homo-multimer is the third unique feature of A3G. The intrinsic propensity of A3G multimerization has been verified by biochemical and structural studies (Jarmuz et al., 2002; Navarro et al., 2005; Burnett and Spearman, 2007; Bennett et al., 2008; Bulliard et al., 2009; Friew et al., 2009; Huthoff et al., 2009; Chelico et al., 2010; McDougall et al., 2011). Because the full-length A3G structure has not been determined, the A3G interface for multimerization remain unclear. However, structural analyses by SAXS (small-angle X-ray scattering), co-immunoprecipitation assays (Wedekind et al., 2006; Bennett et al., 2008), and X-ray crystallography (Shandilya et al., 2010) have demonstrated an interaction between the A3G CTDs (tail–tail), as illustrated in Figure 1C. In addition, homo-dimerization through the NTDs also occurs (head–head), as shown in Figure 1C, and this interaction appears to depend on the presence of RNA (Friew et al., 2009; Huthoff et al., 2009). These observations were supported by an analytical ultracentrifugation study that showed a predominant dimer form of A3G in equilibrium with minor monomeric and tetrameric species under RNA-depleted conditions (Salter et al., 2009).

Chelico et al. (2006, 2008, 2010) have used an *in vitro* system to demonstrate that A3G has a 3′ to 5′ catalytic orientation specificity for the deamination of naked ssDNA (Feng and Chelico, 2011). The preferred asymmetric direction for A3G catalysis likely yields an approximately 30-nt “dead” zone located at the 3′ end of ssDNA that is much less efficiently deaminated by A3G (Chelico et al., 2008, 2010). However, we need further investigations on how significant the length of dead zone is because the central CCC motif of ~40-nt ssDNA can be deaminated efficiently by A3G in our or the other *in vitro* deamination assay systems (Beale et al., 2004; Yu et al., 2004; Iwatani et al., 2006). The formation of tetramers and higher-order homo-oligomers of A3G on ssDNA is required for efficient deamination (McDougall et al., 2011).

## Antiviral Mechanisms of A3G Against *vif*-Deficient HIV-1

**Figure 2:**
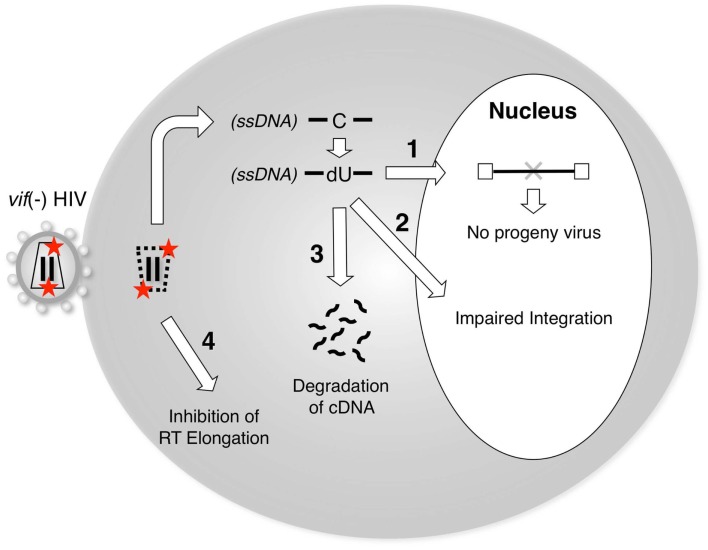
**A3G blocks the reverse transcription and/or integration of *vif*-deficient HIV-1**. Packaging of A3G proteins into *vif*-deficient virus particles is prerequisite for the inhibition of viral replication by A3G. Upon the infection of target cells, A3G blocks the post-entry step of viral replication by one or more of the following mechanisms: (1) Cytidine deamination of nascent reverse transcripts by A3G enzymes could prevent progeny virus production due to inactivating mutations in viral genes and/or proteins. (2) A3G-mediated editing might create aberrant structures at viral DNA ends, which might be inefficient substrates for integration. (3) The reverse transcripts containing dU might induce DNA degradation by cellular DNA repair pathways. (4) RT-mediated elongation could be blocked by the presence of A3G on RNA or DNA templates. A3G might exert both deaminase activity-dependent (1–3) and deaminase activity-independent (4) functions to inhibit *vif*-deficient HIV-1 replication.

A3G exerts its inhibitory activity by being encapsidated into virus particles of *vif*-deficient HIV-1. During the subsequent infection cycle, A3G has been proposed to interfere with reverse transcription and/or integration through one or more molecular mechanisms (Figure 2). Based on whether the enzymatic activity is involved or not, there are two separable mechanisms, i.e., deaminase-dependent and -independent mechanisms.

Although the catalytic center of A3G is clearly critical for its antiviral effect (Mangeat et al., 2003; Navarro et al., 2005; Iwatani et al., 2006; Browne et al., 2009), the precise molecular mechanisms underlying the inhibition of the further processing of A3G-deaminated DNA products in cells remain unclear. A3G-mediated hypermutation of viral genomes is clearly detrimental to further spreading the infection because mutations in the viral structural and/or the regulatory genes may trigger defects in the production of infectious progeny virus (“1” in Figure 2). For example, because the preferred sequences of A3G include TGG (a codon for tryptophan within the viral *orf*), many G-to-A mutations may incidentally produce premature stop codons, such as TAG (or TGA), resulting in viral inactivation (Simon et al., 2005; Pace et al., 2006).

In its second mechanism, A3G reduces the efficiency and specificity of primer tRNA processing and removal, resulting in proviral DNA ends that are aberrant substrates for integration and plus-strand DNA transfer (Luo et al., 2007; Mbisa et al., 2007; “2” in Figure 2). In this mechanism, the presumed deamination sites are located at the plus primer-binding site (PBS), which is annealed by the tRNA. Considering the biochemical characteristics of A3G, it remains unclear how the A3G enzyme deaminates cytidine residues on the DNA/RNA duplex and near the 3′ end of the plus-strand transfer donor DNA, which is supposed to be a presumed “dead” zone for A3G-mediated deamination (Chelico et al., 2008, 2010).

It was hypothesized that the antiviral functions of A3G might be associated with the uracilation of the nascent reverse transcripts (Harris et al., 2003a; Zhang et al., 2003), resulting in their degradation through the activity of cellular DNA glycosylases, e.g., UDG2 (uracil DNA glycosidase 2) and SMUG1 (single-strand selective monofunctional uracil DNA glycosylase). However, several groups have revealed that neither uracil DNA glycosidase affected the antiviral effect of A3G (Kaiser and Emerman, 2006; Mbisa et al., 2007; Langlois and Neuberger, 2008), although one study showed that UDG2 is involved in the degradation of nascent reverse transcripts (Yang et al., 2007). In addition, we cannot exclude the possibility that other unidentified DNA repair enzymes might participate in the degradation mechanism. Therefore, further studies will be required to elucidate the potential factors that precede the degradation of uracilated DNA following A3G-mediated deamination (“4” in Figure 2).

Earlier studies on A3G suggested that G-to-A hypermutation resulting in lethal mutations was the sole basis of the A3G antiviral mechanism. However, more recent studies have demonstrated that the catalytic activity of A3G may not wholly determine its molecular mechanism, i.e., a deaminase–independent mechanism might also be involved in A3G antiviral activity: (i) mutations of the catalytic center do not completely abolish antiviral activity against HIV-1 (Navarro et al., 2005; Iwatani et al., 2006; Holmes et al., 2007; Luo et al., 2007; Miyagi et al., 2008); (ii) A3G inhibits replication of hepatitis B virus without detecting significant G-to-A hypermutation (Turelli et al., 2004; Bonvin and Greeve, 2007; Nguyen et al., 2007; reviewed in Bonvin and Greeve, 2008); and (iii) other A3 proteins block the replication of HIV-1 (Luo et al., 2007; Miyagi et al., 2010), mouse mammary tumor virus (Okeoma et al., 2007), murine leukemia virus (Takeda et al., 2008), and parvovirus adeno-associated virus (Narvaiza et al., 2009) and retrotransposition of LINE-1 and Alu (Bogerd et al., 2006; Muckenfuss et al., 2006; Stenglein and Harris, 2006; Wissing et al., 2011) despite the absence of editing activity.

Several groups have reported that the deaminase-independent mechanisms of reverse transcription inhibition would involve interference with tRNA primer annealing, initiation and elongation of DNA synthesis, and minus-/plus-strand transfer reactions (Guo et al., 2006, 2007; Iwatani et al., 2007; Li et al., 2007; Luo et al., 2007; Mbisa et al., 2007; Anderson and Hope, 2008; Bishop et al., 2008; Zhang et al., 2008). Using enzymatically active recombinant A3G and the *in vitro* reconstituted systems of HIV-1 reverse transcription, it has been demonstrated that A3G blocks all RT-catalyzed DNA elongation processes in a deaminase-independent manner, although the protein does not significantly interfere with tRNA primer placement (Iwatani et al., 2007). Moreover, the analysis of endogenous reverse transcription in cell-free HIV-1 particles also indicated that A3G reduces HIV-1 viral DNA levels by inhibiting the elongation of reverse transcription rather than by inducing the degradation of the reverse transcripts (Bishop et al., 2008). The block of RT elongation by A3G might be attributed to A3G’s unique nucleic acid-binding ability (Iwatani et al., 2007). More recently, Wang et al. (2012) have observed physiological and functional interactions between RT and A3G, although our group has been unable to detect direct interactions using recombinant A3G and RT proteins (Iwatani, Y., and Levin, J. G., unpublished observations). It might be interesting to know whether direct interaction is applicable to other A3 proteins and/or retroviral RTs, i.e., how the broad range of A3G’s inhibitory effect can be linked to the specific interaction between RT and A3G. Further investigations are required to understand the molecular mechanisms of the deaminase-independent pathway in more detail.

## Conclusions

Studies over the past 10 years have established that human APOBEC3 family proteins potently restrict retroviral replication. However, the molecular mechanisms of the A3 family’s antiviral activities remain unclear. Recent biochemical studies of A3G may provide a better understanding of these mechanisms. Currently, it is possible that the deaminase activity of A3G is largely required for its antiviral activity against v*if*-deficient HIV-1, although it is not known whether A3G-mediated deamination and/or the architecture of the catalytic center in A3G are intrinsically required for its inhibitory activity. Further investigations will provide the fundamental answers to explain the first observation by von Schwedler et al., made when A3G had not yet been discovered.

## Conflict of Interest Statement

The authors declare that the research was conducted in the absence of any commercial or financial relationships that could be construed as a potential conflict of interest.
